# Effects of continuity of maternal health services on immediate newborn care practices, Northwestern Ethiopia: multilevel and propensity score matching (PSM) modeling

**DOI:** 10.1016/j.heliyon.2022.e12020

**Published:** 2022-12-02

**Authors:** Muluwas Amentie Zelka, Alemayehu Worku Yalew, Gurmesa Tura Debelew

**Affiliations:** a Department of Public Health, College of Health Sciences, Assosa University, Assosa, Ethiopia; b Department of Reproductive Health, School of Public Health, College of Health Sciences, Addis Ababa University, Addis Ababa, Ethiopia; c Department of Biostatistics and Epidemiology, School of Public Health, College of Health Sciences, Addis Ababa University, Addis Ababa, Ethiopia; d Department of Population and Family Health, Institute of Health, Jimma University, Jimma, Ethiopia

**Keywords:** Benishangul Gumuz region, Continuum of care, Maternal health, Newborn care, Practice

## Abstract

**Background:**

Despite priority being given to maternal and child health programs in Ethiopia, the reduction of neonatal mortality rate is stagnant, which is more than double the national target. Immediate newborn care and continuity of maternal health services are comprehensive, wide-ranging, and core strategies to overcome neonatal mortality and morbidity. However, the evidence of immediate newborn care practices and the effectiveness of continuity of maternal health services on immediate newborn care practices are scarce. Hence, this study aimed to fill this gap.

**Methods:**

A prospective follow-up study was conducted from March 2020 to January 2021, among 2198 pregnant women screened from the study areas. The data were collected using pretested semi-structured questionnaires and a registration logbook. Data were coded, entered, cleaned, and analyzed using STATA software 14. Descriptive statistics, multilevel regression, and propensity score matching (PSM) models were computed. Finally, ICC (ρ), AOR, and coefficient (β) along with 95%CI were calculated and statistical significance was considered at a p-value < 0.05.

**Results:**

The magnitude of immediate newborn care practice was 50.9% (95%CI: 50.5%, 51.3%). Partner attended primary cycle school (AOR = 2.32), women attended ANC visit ≥4 (AOR = 2.69), initiated 1st ANC visit between 4–6 months of GA (AOR = 0.47), IFA supplementation (AOR = 2.99), women who make a decision (AOR = 2.25), women whose husband make a decision (AOR = 1.66) and immunizing the newborn (AOR = 2.46) were determinant factors of immediate newborn care practices. As treatment effect, completion of COC in MHS via time dimension (β = 0.31; 95%CI: 0.27, 0.35); whole key service MHS (β = 0.43; 95%CI: 0.39, 0.48) and COC via space dimension (β = 0.17; 95%CI: 0.12, 0.21) were significantly increased the likelihood of immediate newborn care practices.

**Conclusion:**

The magnitude of optimal immediate newborn care practices was low. Different enabling factors were discovered in the study. Therefore, strengthening those enabling factors such as partner education, immunization program, IFA supplementation, early initiation and receiving ANC services, the decision-making power of women and partners, as well as scaling up a continuum of care in maternal health services are strongly recommended.

## 1 Introduction

Globally, the neonatal mortality rate fell from 36 deaths in 1990 to 19 deaths in 2015, and the number of neonatal deaths declined from 5.1 million to 2.7 million [1]. Based on this evidence, the neonatal mortality rate has decreased at a slower rate than post-neonatal mortality [47% vs. 58%]. Most of the deaths are caused by diseases that are easily preventable or treatable with proven and cost-effective interventions [1]. The main direct causes of neonatal death are preterm birth, severe infections, asphyxia, and neonatal tetanus [2, 3]. Even though low birth weight is a substantial indirect cause of newborn death, poverty and maternal complications during pregnancy, childbirth, and after childbirth are significant risk factors [4, 5]. As a result of those challenges, every hour, 450 newborn children die, mainly from preventable causes, which is intolerable during the 21st century [6].

According to World Health Organization (WHO) recommendation, essential newborn care is a comprehensive, wide range and core strategy to overcome neonatal mortality and morbidity [7, 8]. The pooled result of nine studies between 2007 and 2017 revealed that neonatal mortality was reduced by 45% and perinatal mortality was reduced by 30%, which is attributed by the proper implementation of essential newborn care interventions [8]. However, neonatal and perinatal mortality in Ethiopia is the highest in the World (NMR 29 death/1000 LB) whereas in Benishangul Gumuz Region neonatal mortality rate was 35 per 1000 live births which is the highest in Ethiopia [9]. However, utilization of essential newborn care is low in Ethiopia: Ethiopia (48.77%) [10]; Southwest (41%) [11]; Northwest (73.8%) [12]; Awi zone (62.7%) [13]; Southern Ethiopia (24%) [7]; Northeast Ethiopia (62.9%) [14]; Western Ethiopia (44.1%) [15] and the factors that obscure practice of essential newborn care are residence area, postnatal care, counseling during pregnancy and delivery, antenatal care follow-up, and maternal educational status, in-service training, knowledge status, availability of drug and medical equipment’s, advice on newborn care, plan pregnancy, supportive supervision, the interest of health providers and availability of vitamin K [7, 10, 11, 12, 13, 14, 15].

Different evidences reveal that status of essential newborn care practices is low and different factors are identified in Ethiopia [7, 8, 10, 11, 12, 13, 14, 15]. Moreover, those studies used a cross-sectional study design and ways of analysis were traditional logistic regression which ignored cluster variation. This results in over or under estimate the status of immediate newborn care practices and determinant factors that obscure the level of practices. Still now, the status of immediate newborn care practices and determinant factors are not clearly understood in the region, and also effects of a continuum of care (COC) in maternal health services on immediate newborn care practice has not been studied in Ethiopia, particularly in Benishangul Gumuz Region. Hence, it is important to have updated information on the magnitude of immediate newborn care practice and determinant factors by considering individual and community-level cluster variation for the improvement of policy and program implementation. Even though we have few cross-sectional studies on the status, studies on the effectiveness of completion of a continuum of care in maternal health services on immediate newborn care practices by strong analytic design are paucity in Ethiopia and not found in the region.

Therefore, this study aimed to determine the effectiveness of a continuum of care in maternal health services on immediate newborn care practices by implementing strong analytic design and analysis. Moreover, the community-level (level - 2) factors and individual-level (level - 1) factors that affect the utilization of immediate newborn care practices were determined. The finding of this study will give a clue for policymakers and program implementers to improve the coverage of essential newborn care practices and effectiveness of a continuum of care in maternal health services and how the nation progresses to achieve the stated SDG target in the reduction of neonatal mortality.

## 2 Materials and methods

### 2.1 Study design and setting

Community and health facility-linked prospective follow-up study design was employed in Benishangul-Gumuz Regional State (BGRS) from March 2020 to January 2021. The region is one of the eleven states of Ethiopia’s Federal Democratic Republic of Ethiopia. Assosa town is the capital city of the region, located 670 kms West of Addis Ababa, the capital city of Ethiopia. The region has three zones (namely *Assosa, Metekel and Kemashi zone*), three town administrative cities (namely *Assosa town, Gilgel Beles town and Kemashi town*), 21 districts/*Woredas*, 1 special district/*Woredas* (namely *Mao-Komo Special Woreda*) and 475 clusters/*Kebeles* (439 rural and 36 urban clusters/*Kebeles*). The region represents around 4.6% of the total land area of Ethiopia and most of the people in the region are sparsely populated [16].

### 2.2 Source population and study participants

All births that were registered as “live births” or “stillbirths” at the time of birth and born from women (registered as pregnant women during the baseline survey) in the Region were considered as source population. Whereas, the study participants were newborns registered as “live birth” or “stillbirth” (as declared by women, birth attendants, or health workers) at the time of birth and pregnant women within the selected districts/*woredas* which were selected by simple sampling techniques.

### 2.3 Sample size and sampling technique

This study aimed to look at the magnitude and determinant factors of immediate newborn care practice. Hence, to determine the sample size, two options of sample size determination were used to take a large sample size. The sample size for the first choice was computed using a single population proportion to look at the level of optimal immediate newborn care practice based on the following assumption: the proportion of newborns who received optimal immediate newborn care practice is 30.8% (p = 0.308) [17]. The margin of error is 5% (d = 0.05) with a 95% confidence interval (1.96) taken into consideration. Then, by taking a design effect of 2 and a non-response rate of 10% into account, the computed sample size was 719 neonates. Similarly, the second option of sample size was calculated to estimate the effect of determinant factors on receiving optimal immediate newborn care practices. Hence, two population proportion formula was employed to estimate the sample size for this study. For all conceivable determinant factors, the sample sizes were calculated. Among all the factors considered, women educational level provided the maximum sample size. The proportion of babies who were born from mothers that attended secondary school received optimal immediate newborn care practices is 29.5% (p_1_ = 0.295) whereas among mothers who not attended formal education is 13.6% (p_2_ = 0.136) [17]; **P** (pooled population proportion) = $\frac{P_{1}+P_{2}}{1+r}$ was calculated (**P** = 0.22); **r** = ratio of exposure to non-exposure pregnant women equal to 1:1; a 95% level of confidence and 80% power, having a design effect of 2 and a non-response rate of 10%. Then, the sample size was ***515*** newborns babies. As a result, the maximum sample size calculated for this study was 719 newborns. Even though this study was part of large research work, the sample size was calculated for another objective found to be 2402 pregnant women [18]. After 11 months of follow-up and excluding abortion cases, the final sample size considered for this study was 2065 babies.

Due to broad settings/areas included in this study, a multistage sampling technique was applied to select the study participants. Primarily, two zones and one town administration city were selected by simple random sampling (SRS) from three zones and three town administrations respectively. Secondly, four districts/“*woredas*” from the Assosa zone, two districts/“*woredas*” from the Metekel zone and two districts/“*woredas*” from the Assosa town administration were selected using simple random sampling (SRS) techniques. Thirdly, among the selected districts/woreda, 51 study clusters such as 7 kebeles from each district/“*woreda*” except Assosa district/“*woreda*” (10 *kebeles*) and five Administrative villages/“*ketenas*” from each district/“*woreda* of town administration were selected using simple random sampling (SRS) techniques. Finally, all pregnant women were enumerated using house-to-house visits in the selected kebeles/administrative villages/“*ketenas*” and all registered pregnant women were included in the study. All women who claimed a pregnancy of eight weeks or more, as defined by the loss of two consecutive menses, were deemed eligible and joined the study. The selected pregnant women were follow-up for an average of 11 months. Besides, the baseline house-to-house surveys, the public health facilities that serve the selected study areas were listed. As result, 46 health facilities (3 hospitals, 12 health centers and 31 health posts) were identified and included in the health facility-based survey.

### 2.4 Data collection process

A semi-structured questionnaire was designed in English which was adapted from EDHS 2016 [9], National Technical Guidance for MPDSR 2017 [19], MCH Program Indicator Survey 2013 [20], tools from a survey conducted in Jimma Zone, Southwest Ethiopia [21], survey tools conducted in rural South Ethiopia [22] and other relevant different literature. Hence, to ensure the quality of data, training, pretest, supervision and use of local languages were made.

### 2.5 Data management and analysis

Data were coded and entered into Epi Info version 7.2.2.6 to control logical mistakes. The data were then cleaned, edited and analyzed using STATA Software version 14. Descriptive statistics were computed for all variables. Bi-variable crude odds ratio and 95% confidence interval (CI) were used to select candidate variables for multivariable analysis (p < 0.25). A maximum likelihood estimate of the independent effect on the outcome variable was calculated at a significant level (P < 0.05). Principal Component Analysis (PCA) was used to compute and categorize the household wealth index. Before running the full model, the coefficient of interaction term was assessed and all included variables had p ≥ 0.1 which indicated that no interaction effect. Similarly, the multi-collinearity effect between independent variables was determined by using variance inflation factors (VIF > 10%). All included variables had VIF <10 which implied that no multi-collinearity effect.

Even though the multistage sampling method was used because of the different levels of factors, a multilevel regression model was used by using STATA 14 to identify factors having significant associations with optimal newborn care practices. Kebeles/Administrative villages/“*Ketenas*” were considered as clusters and cluster level variables: place of residence, access to a health center and household wealth index were taken as higher level (*level – 2*). Even though pregnant women were nested within the community, individual-level variables such as socio-demographic, obstetric, information and maternal health services were taken as lower level (*level – 1*). The goodness of fit of the multilevel model was tested by the log-likelihood ratio (LR) test and found to be statistically significant so that data fit the model. Finally, propensity score matching was applied to estimate the effect of a continuum of care in maternal health services on optimal newborn care practices.

### 2.6 Ethical clearance

This study was endorsed after obtaining ethical approval from concerned bodies. Ethical approval was obtained from the Research Review and Ethics Committee (REC) of the School of Public Health, College of Health Sciences, Addis Ababa University with protocol number SPH/3089/011 and the Institutional Review Board (IRB) of College of Health Sciences, Addis Ababa University with protocol number 048/19/SPH. The Benishangul Gumuz Regional Health Bureau provided legal approval letters to their respective local administrations before approaching study participants as well as local administrations. Written informed consent, as well as verbal consent, was obtained from each respondent before actual data collection and confidentiality of the data was strictly maintained.

## 3 Results

### 3.1 Status of immediate newborn care practices

**Figure 1:**
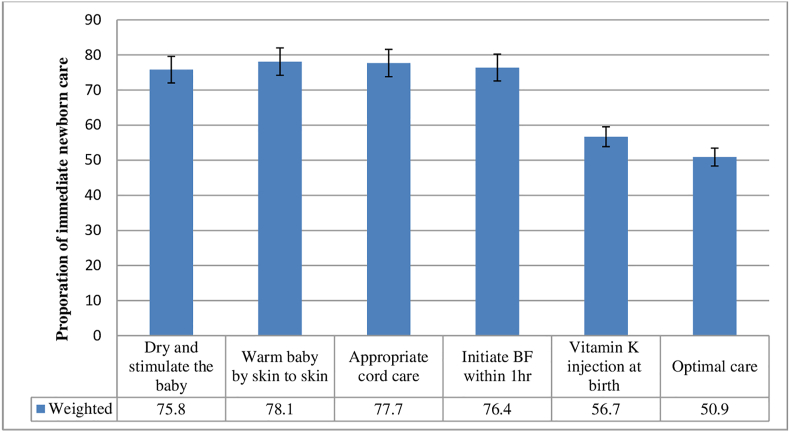
Utilization of immediate newborn care for the study subject in Benishangul Gumuz region, Northwestern Ethiopia, March 2020–January 2021.

Among the study participants, a total of 2065 live births and stillbirths were considered for analysis to determine the status of immediate newborn care practices and associated factors. Among components of immediate newborn care practices, 1593 (75.8%), 1690 (78.1%), 1666 (77.7%), 1644 (76.4%) and 1258 (56.7%) practiced dry and stimulate the baby, keep the baby warm by skin to skin contact, appropriate cord care, initiate breastfeeding within 1 h after birth and vitamin K injection at birth respectively. Then, using those parameters, a composite index was created by using PCA and the mean score was determined. Thus, 50.9% (95%CI: 50.5%, 51.3%) of neonates scored above or equal to the mean score and were categorized as optimal immediate newborn care practices (Figure 1).

### 3.2 Determinants of immediate newborn care practices

The factors affecting immediate newborn care practices at the community and individual levels were identified by using a multilevel model. Before running the full model, ICC (ρ) was calculated in the empty model for the outcome to decide whether the data fit a multilevel regression model or not. Then, ICC (ρ) was calculated using the full model to detect the variability attributed by clusters after controlling the individual level.

For immediate newborn care practice outcome, ICC (ρ) was calculated in the empty model and it was found to be 0.60, indicating that 60% of the variation was contributed by cluster variations. The test of preference for log-likelihood *versus* logistic regression was also statistically significant (p < 0.0001). Then, the full model was run by including both the cluster-level and individual-level variables, and the ICC (ρ) was reduced to 0.55. This again indicated that 55% of the variation was attributed to cluster-level variables. The preference for log-likelihood *versus* logistic regression was again strongly statistically significant (p < 0.00001). Hence, this suggests that using multilevel analysis is more appropriate and accurate than the ordinary binary logistic regression model (Table 1).

**Table 1:** Parameters, odds ratio and Test of Goodness-of-fit of the Mixed-effects Models in Benishangul Gumuz Region, Northwest Ethiopia, 2021.

*Models*	Fixed intercept -cons(95%CI)	Random effect as Level-2 variance var(-cons (95%CI))	Intra-class Correlation Coefficient: ICC(ρ)	Log likelihood (LR)-deviance	Significance of LR test Vs Logistic regression (P-value)
**Newborn care practices**∗					
*Empty model*	0.97(0.52, 1.79)	5.0(3.1, 8.08)	0.60 = 60%	-979.2	p < 0.0001
*Full model*	1.59(0.11, 0.52)	4.1(2.48, 6.64)	0.55 = 55%	-826.48	p < 0.0001

P value less than 0.05 is statistically significant and the data fit for the multilevel model.

∗ Multilevel regression model applied to measure the effect of factors on this outcome.

After adjusting for confounding effects, a multivariable multilevel model was performed. Then, the determinants of immediate newborn care practices were assessed at cluster and individual level factors. Despite this, the cluster level (level – 2) factors weren’t found to have a statistically significant association with immediate newborn care practices. However, at the individual level (*level - 1*) factors were found to have statistically significant association with immediate newborn care practices.

The odds of having optimal immediate newborn care practices among women whose partners attended primary cycle school (AOR = 2.32; 5%CI: 1.14, 4.75), high school (AOR = 2.67; 5%CI: 1.26, 5.66) and tertiary education (AOR = 2.20; 5%CI: 1.06, 4.56) were two times higher than among those whose partners had no formal education. Similarly, the odds of receiving optimal immediate newborn care practices among women who received the recommended ANC visit (≥4 visits) (AOR = 2.69; 5% CI: 1.56, 4.64) and iron and folic acid (IFA) supplementation during pregnancy (AOR = 2.99; 95%CI: 1.60, 5.57) were three-fold higher than among their counterpart. Moreover, the odds of practicing optimal immediate newborn care among women who had a decision-making power on health services seeking (AOR = 2.25; 95%CI: 1.23, 4.13) and women whose husband decided on health services seeking (AOR = 1.66; 95%CI: 1.04, 2.67) were higher than among women within their counterpart.

However, the odds of practicing optimal immediate newborn care among women initiated a 1st ANC visit between 4 and 6 months of gestational age (AOR = 0.47; 95%CI: 0.27, 0.82) and after 6 months of gestational age (AOR = 0.11; 95%CI: 0.03, 0.39) were 53% and 73% respectively were lower than among women who started 1st ANC visit within 3 months of gestational age (Table 2).

**Table 2:** Multilevel Models analysis on determinant factors associated with immediate newborn care practices in Benishangul Gumuz Region, Northwest Ethiopia 2021.

Factors	Newborn care practices		Crud OR 95%CI	Adjusted OR 95%CI
	Suboptimal	Optimal		
**Level – 2 (Community level) variables**				
**Place of Residents**				
Urban	369 (51.46)	348 (48.54)	1	1
Rural	588 (43.62)	760 (56.38)	0.80(0.35, 1.85)	0.97 (0.26, 3.56)
**Time takes to reach HC**				
<2 Hours	680 (44.01)	865 (55.99)	1	1
≥2 Hours	277 (53.27)	243 (46.73)	**0.46 (0.28, 0.78)**	0.61 (0.27, 1.38)
**Leve-1 (individual level) variables: socio-demographic characteristic**				
**Household wealth index**				
1st Quintile (Poor)	310 (44.86)	381 (55.14)	1	1
2nd Quintile (Middle)	308 (43.69)	397 (56.31)	1.33(0.84, 2.11)	1.33(0.79, 2.23)
3rd Quintile (Rich)	339 (50.67)	330 (49.33)	0.92(0.56, 1.49)	1.28(0.55, 2.95)
**Age (Years)**				
<20	78 (49.06)	81 (50.94)	1	1
20–29	582 (44.09)	738 (55.91)	1.33 (0.84, 2.11)	0.26 (0.04, 1.75)
≥ 30	297 (50.68)	289 (49.32)	0.92 (0.56, 1.49**)**	0.24 (0.04, 1.61)
**Ethnicity**				
Berta	416 (37.99)	679 (62.01)	1	1
Others	541 (55.77)	429 (44.23)	0.68 (0.43, 1.08)	0.67 (0.29, 1.52)
**Woman education level**				
Formal education	626 (48.75)	658 (51.25)	1	1
Primary school	161 (41.60)	226 (58.40)	1.36 (0.97, 1.91)	1.46 (0.76, 2.79)
High School	91 (42.13)	125 (57.87)	1.40 (0.93, 2.11)	0.59 (0.27, 1.33)
Tertiary education	79 (44.38)	99 (55.62)	**1.59 (1.03, 2.45)**	1.27 (0.53, 3.02)
**Partner education level**				
Formal education	575 (49.65)	583 (50.35)	1	**1**
Primary school	121 (50.0)	121 (50.0)	**1.56 (1.03, 2.37)**	**2.32 (1.14, 4.75)**
High School	112 (40.43)	165 (59.57)	**2.06 (1.39, 3.06)**	**2.67 (1.26, 5.66)**
Tertiary education	128 (38.91)	201 (61.09)	**2.24 (1.56, 3.22)**	**2.20 (1.06, 4.56)**
**Age at first marriage**				
<18	377 (52.65)	339 (47.35)	1	1
≥ 18	580 (42.99)	769 (57.01)	1.23(0.95, 1.60)	1.54(0.81, 2.90)
**Age at first pregnancy**				
<19	388 (52.15)	356 (47.85)	1	1
≥19	569 (43.07)	752 (56.93)	1.67(0.89, 1.52)	0.72(0.38, 1.35)
**Stillbirth ever had**				
No	663 (47.32)	738 (52.68)	1	1
Yes	84 (49.12)	87 (50.88)	0.76(0.49, 1.18)	0.85(0.47, 1.58)
**Place of delivery for previous delivery**				
Home	249 (61.03)	159 (38.97)	1	1
Health post	131 (37.22)	221 (62.78)	**2.36(1.43, 3.88)**	1.15(0.58, 2.28)
Health center	279 (45.66)	332 (54.34)	**1.84(1.24, 2.74)**	0.78(0.40, 1.53)
Hospital	88 (43.78)	113 (56.22)	**2.11(1.15, 3.87)**	0.67(0.28, 1.61)
**Pregnant related problem during previous pregnant**				
No	607 (47.76)	664(52.24)	1	1
Yes	140 (46.51)	161 (53.49)	1.35(0.94, 1.92)	1.07(0.04, 8.59)
**Availability of maternal health services**				
No	74 (60.16)	49 (39.84)	1	1
Yes	883 (45.47)	1059 (54.53)	**1.99(1.18, 3.35)**	0.58(0.04, 8.59)
**Provision of maternal health services**				
No	90 (63.38)	52 (36.62)	1	1
Yes	867 (45.09)	1056 (54.91)	**2.59(1.59, 4.23)**	4.76(0.37, 60.47)
**Time of 1st ANC initiation**				
1–3 months of GA	247 (45.66)	294 (54.34)	1	**1**
4–6 months of GA	539 (40.71)	785 (59.29)	**0.50(0.36, 0.71)**	**0.47(0.27, 0.82)**
After 6 months of GA	114 (87.02)	17 (12.98)	**0.06(0.03, 0.13)**	**0.11(0.03, 0.39)**
**Number of ANC visit**				
<4	422 (68.73)	192 (31.27)	1	**1**
≥ 4	535 (36.87)	916 (63.13)	**4.95(3.65, 6.71)**	**2.69(1.56, 4.64)**
**IFA supplementation**				
No	314 (80.10)	78 (19.90)	1	1
Yes	643 (38.43)	1030 (61.57)	**4.84(3.47, 6.76)**	**2.99(1.60, 5.57)**
**TT for pregnancy**				
No	334 (66.01)	172 (33.99)	1	1
Yes	623 (39.96)	936 (60.04)	**3.40(2.51, 4.60)**	1.11(0.63, 1.94)
**Information offered on facility delivery**				
No	97 (59.15)	67 (40.85)	1	1
Yes	860 (45.24)	1041 (54.76)	1.51(0.98, 2.33)	0.61(0.27, 1.40)
**Pregnant related problems during labour of last delivery**				
No	786 (44.43)	983 (55.57)	1	1
Yes	170 (57.63)	125 (42.37)	**0.57(0.42, 0.79)**	0.93(0.46, 1.88)
**Women make a decision**				
No	335 (67.40)	162 (32.60)	1	**1**
Yes	622 (39.67)	946 (60.33)	**2.72(1.95, 3.81)**	**2.25(1.23, 4.13)**
**Husband make a decision**				
No	418 (44.33)	525 (55.67)	1	**1**
Yes	539 (48.04)	583 (51.96)	1.29(0.99, 1.69)	**1.66(1.04, 2.67)**
**Attendant of delivery care for current pregnancy**				
Unskilled provider	431(54.9)	354(45.1)	1	1
Skilled provider	526(41.09)	754(58.91)	**1.86(1.44, 2.39)**	1.13(0.67, 1.89)
**Duration of labour**				
Less than 12	657 (45.09)	800 (54.91)	1	1
B/n 12–24 h	225 (49.13)	233 (50.87)	**0.54(0.40, 0.74)**	0.65(0.39, 1.09)
Greater than 24 h	75 (50.0)	75 (50.0)	0.89(0.58, 1.37)	1.06(0.41, 2.72)
**Pregnant related problem immediately after delivery**				
No	833 (44.62)	1034 (55.38)	1	1
Yes	124 (62.63)	74 (37.37)	**0.39(0.26, 0.57)**	0.49(0.23, 1.06)
**Gestational age at birth**				
Preterm *(< 37 weeks*)	105 (43.75)	135 (56.25)	1	1
Term *(≥ 37 weeks*)	851 (46.66)	973 (53.34)	**1.47(1.01, 2.12)**	0.84(0.45, 1.55)
**Time of premature rupture of membrane before labour**				
< =1 h	325 (39.93)	489 (60.07)	1	1
1–12 h	575 (50.80)	557 (49.20)	**0.74(0.56, 0.97)**	1.31(0.84, 2.01)
>12 h	48 (51.06)	46 (48.94)	**0.50(0.28, 0.91)**	2.67(0.93, 7.68)
**Birth weight**				
Abnormal	130 (48.33)	139 (51.67)	1	1
Normal	691 (41.68)	967 (58.32)	**1.62(1.15, 2.28)**	1.36(0.76, 2.43)

### 3.3 Effect of continuum of care on immediate newborn care practices

A propensity score matching (PSM) model was used to minimize or reduce the confounding effect of covariate when estimating the treatment effect on the outcome variable. Of the five different approaches of propensity score matching (PSM), nearest neighbor matching particularly one-to-one matching was used to estimate the effects of the completion of a continuum of care in maternal health services via both time and space dimensions on immediate newborn care practices. After matching treated and controlled individuals, women who completed continuity of maternal health services via both dimensions were more likely to receive optimal immediate newborn care practices as compared to those who discontinued the services. The validity of the finding from propensity score matching analysis is tested using standardized mean difference (SMD) and variance ratio (VR). Hence, the model diagnostic reveals that SMD < 0.1 which declares that there is a balance between the treat and control group, as well as VR = 1 which implies that the matched sample is a good match.

Therefore, the average treatment effect of a continuum of care in maternal health services found to be received 1st ANC visit (β = 0.27 95%CI: 0.19, 0.3; p < 0.001); completed 4th ANC visit (β = 0.31; 95%CI: 0.25, 0.36; p < 0.001); skilled attendant of delivery services (β = 0.14; 95%CI: 0.1, 0.19; p < 0.001); completed continuity of care for both 4th ANC and skilled delivery (β = 0.26; 95%CI: 0.22, 0.3; p < 0.001); completion of continuum of care in maternal health services via time dimension (β = 0.31; 95%CI: 0.27, 0.35; p < 0.001); completed key services of ANC package (β = 0.4; 95%CI: 0.36, 0.45; p < 0.001), completion of key services of PNC package (β = 0.39; 95%CI: 0.35, 0.43; p < 0.001), complete whole key service maternal health services (β = 0.43; 95%CI: 0.39, 0.48; p < 0.001) and completed continuum of care via space dimension (β = 0.17; 95%CI: 0.12, 0.21; p < 0.001) were associated with a significantly increasing utilization of immediate newborn care practice (Table 3).

**Table 3:** Propensity score matching analysis on effect of completion of continuum of care in maternal health services on immediate newborn care practices in Benishangul Gumuz region, Northwest Ethiopia 2021.

Factors	Immediate newborn care		ATE		ATET	
	Suboptimal	Optimal	β 95%CI	P – value	β 95%CI	P – value
**I. Continuity of care in maternal health services via time dimension**						
**First ANC services**						
No Received	127 (69.40)	56 (30.60)	**0.27 (0.19, 0.3)**	P < 0.001	**0.27 (0.19, 0.35)**	P < 0.001
Received	830 (44.10)	1052(55.9)				
**Fourth ANC services**						
Discontinued	422 (68.73)	192 (31.12)	**0.31 (0.25, 0.36)**	P < 0.001	**0.3 (0.23, 0.36)**	P < 0.001
Completed care	535 (36.87)	916 (63.13)				
**Delivery care Services**						
Unskilled delivery services	431 (54.90)	354 (45.10)	**0.14 (0.1, 0.19)**	P < 0.001	**0.13 (0.08, 0.18)**	P < 0.001
Skilled delivery services	526 (41.09)	754 (58.91)				
**Continuity of care for both 4th ANC and Skilled delivery**						
Discontinuity of car	624 (57.46)	462 (42.54)	**0.26(0.22, 0.3)**	P < 0.001	**0.26 (0.21, 0.3)**	P < 0.001
Completion of car	333 (34.01)	646 (65.99)				
**Completion of continuity of maternal health services via time dimension**						
Discontinuity of COC	765 (57.35)	569 (42.65)	**0.31 (0.27, 0.35)**	P < 0.001	**0.29 (0.24, 0.33)**	P < 0.001
Completion of COC	192 (26.27)	539 (73.73)				
**II. Continuity of care for key maternal health services**						
**Continuity of key services of ANC package**						
Discontinuity of key services	644 (69.70)	280 (30.30)	**0.4 (0.36, 0.45)**	P < 0.001	**0.4 (0.35, 0.45)**	P < 0.001
Completion of key services	313 (27.43)	828 (72.57)				
**Continuity of key services of PNC package**						
Discontinuity of key services	732 (58.94)	510 (41.06)	**0.39 (0.35, 0.43)**	P < 0.001	**0.38 (0.34, 0.42)**	P < 0.001
Completion of key services	225 (27.34)	598 (72.66)				
**Continuity of key services of all key services of MHS package**						
Discontinuity of key services	867 (53.99)	739 (46.01)	**0.43 (0.39, 0.48)**	P < 0.001	0.42 (0.38, 0.46)	P < 0.001
Completion of key services	90 (19.61)	369 (80.39)				
**III. Continuity of care for maternal health services via space dimension**						
Discontinuity of COC	656 (52.61)	591 (47.39)	**0.17 (0.12, 0.21)**	P < 0.001	**0.12 (0.07, 0.17)**	P < 0.001
Completion of COC	301 (36.80)					

**The bold values** indicate statistically significant association (p < 0.05).

After matching treated and control group; average treatment effect of completion of a continuum of care in maternal health services via time dimension were increasing uptake of immediate newborn care practices by *0.29* as compared with discontinued maternal health services among the treated group(β = 0.29; 95%CI: 0.24, 0.33). Similarly, average treatment effect of completion of continuum of care in maternal health services via space dimension were increasing utilization of immediate newborn care practices by *0.12* as compared with discontinued maternal health services among the treated group (β = 0.12; 95%CI: 0.07, 0.17). In other words, women who completed continuum of care in maternal health services had more practiced immediate newborn care than those who that did discontinued the services among the treated group (Table 3).

## 4 Discussion

### 4.1 Status of immediate newborn care practices

The recommended immediate newborn care practices are dry and stimulate the baby, warm the baby by skin-to-skin contact, appropriate cord care, initiate breastfeeding within 1 h and administer vitamin k at birth [23]. Hence, the overall status of immediate newborn care practices was determined using composite indicators which protect newborns from illness and death. As result, the level of optimal immediate newborn care practices was 50.9% which was consistent with studies in Ethiopia (48.77%) [10]; Ethiopia (47%) [24], Senegal (48%) [24], Democratic Republic of Congo (49%) [24], Zambia (49%) [24], Benin (50%) [24], Mali (51%) [24] and Uganda (52%) [24].

However, the finding of the current study is lower than evidences from Awi zone (62.7%) [13]; Northeast Ethiopia (73.8%) [14]; Nekemte city (78.7%) [15]; Tigray region (66.93%) [10], Amhara region (59.62%) [10], North Gondar zone (66.3%) [25], Northwestern zone of Tigray (59.8%) [26], Mozambique (76%) [24], Eritrea (78%) [24] and Malawi (95%) [24], but higher than findings of studies in Southern Ethiopia (24%) [7]; Western Ethiopia (44.1%) [15]; Southwest Ethiopia (41.0%) [11], SNNPR region (32.15%) [10], Nekemte city (44.1%) [15]; Chancho district (38.4%) [27], Hossana town (31%) [28], Bahar Dar city (13.7%) [29], Enderta Tigray Region (40.7%) [30], Aksum town (26.7%) [31], pooled estimate of East Africa (38%) [32] and Gedeo district (24.1%) [33], Guinea (17%) [24], Ivory Coast (31%) [24], Nigeria (33%) [24] and Chad (34%) [24]. Generally, the coverage of optimal immediate newborn care practice is low, but those interventions are crucial for the survival of the newborn by reducing early neonatal death and illness. This discrepancy in the results within the country and outside the nation is due to low awareness of mothers on neonatal problems and the importance of neonatal care that might have led to low utilization of the services and also socio-cultural variability, different study design, times and place.

In this study, the researcher found that practicing dry and/or stimulating the baby was 75.8% which is consistent with studies done in Southwest Ethiopia (80.8%) [11] and Southern Ethiopia (80.4%) [7]. However, it is lower than studies done in the Northwest zone of Tigray region (83.8%) [26], Hossana town (82.2%) [28], Chancho district (95.4%) [27] and Northeast Ethiopia (99.2%) [14] but higher than evidence from Awi zone (55.2%) [13]. This variation could be due to a lack of knowledge and awareness on the advantage of newborn care practice among women and the presence of home delivery in the study area which contributes to a low level of practice.

World Health Organization (WHO) recommends newborns should be wrapped during the assessment, suction and ventilate to be protected from heat loss and also gain heat from the mother through skin-to-skin contact [23, 34]. It is essential to promote the health of newborn babies [34]. Besides, this study found that 78.1% of mothers were keeping their babies warm through skin-to-skin contact, which is lower than studies conducted in Northeast Ethiopia (100%) [14], Arba Minch district (93.8%) [35], Bahar Dar city (85.2%) [29] and Northwest zone of Tigray region (84.4%) [26]; whereas, it is higher than studies done in Aksum town (32.6%) [31], Hossana town (30.6%) [28]; North Gondar (66.3%) [25]; Chancho district (71.0%) [27]; rural areas of Northern Ghana (5.2%) [36], Enderta district (67.7%) [30], Hossana town (69.4%) [28]; rural Sidama zone (52%) [37]; Awi zone (48.8%) [13], Southern Ethiopia (55.3%) [7] and Ghana (46.8%) [38]. This discrepancy could be due to the time of study and the variability of socio-economic factors.

Appropriate cord care is one of the WHO Initiatives in immediate newborn care which looks to improve hygiene and infection control practices at the time of birth [23]. Hence, this study revealed that appropriate cord care is 77.7%, which is similar to studies done in North Gondar (75.4%) [25] and the Rural Sidama zone (73%) [37]. However, it is lower than findings from rural areas of Northern Ghana (90.8%) [36], Arba-Minch (92.0%) [35] and Southern Ethiopia (96.4%) [7] but higher than evidence from the Awi zone (53.2%) [13], Aksum town (42.8%) [31]; Hossana town (32.9%) [28]; Bahar Dar city (14.8%) [29], Chancho district (52.9%) [27] and Nekemte city (68.3%) [15]. This discrepancy may be the variation of cultural practice and belief in caring umbilical cord. To some extent, traditional practice on the umbilical cord in the study area is lower as compared with the town areas. This variation could be due to the presence of community-based interventions by health extension workers (HEWs).

Breastfeeding should be started as soon as possible for a variety of reasons. Early suckling benefits mothers by stimulating breast milk production and facilitating the release of oxytocin, which aids uterine contractions and lowers postpartum blood loss [23, 39]. Besides these, this study found that 76.4% of mothers initiate breastfeeding within 1 h after birth which is consistent with the findings in rural areas of Northern Ghana (73.5%) [36], Nekemte city (77.2%) [15], rural Sidama zone in Southern Ethiopia (78%) [37], North Gondar-Northwest Ethiopia (72.2%) [25]; Enderta district (81.7%) [30] and Southwest Ethiopia (81.7%) [11]. This discrepancy may be due to the knowledge and awareness gap on the importance of colostrum for neonatal health in the study areas.

However, the finding in this study is lower than studies done in Bihar, India (97.7%) [40]; Northwest (83.8%) [26]; Bahar Dar city (87.8%) [29]; Hossana town (84%) [28] and Northeast Ethiopia (100%) [14] but it is higher than evidence from Ghana (46.8%) [38], Arba Minch (66.3%) [35], Chancho district (59.8%) [27], Aksum town (63.1%) [31]; Southern Ethiopia (45.8%) [7] and Awi zone (82.1%) [13]. The disparity between the results could be explained by differences in study techniques and locations, socio-economic and demographic characteristics of study participants, and the availability and accessibility of health infrastructures.

Vitamin K is an important component of immediate newborn care because it aids in blood clotting and prevents major bleeding [23, 41]. During pregnancy and breastfeeding, the newborns do not receive enough amount of vitamin K from their mothers [41]. As result, newborns are at risk of developing a rare condition known as vitamin K deficiency hemorrhage, which is potentially fatal [41, 42]. Hence, this study explored that vitamin K injection at birth is 56.7% which is extremely lower than evidence from Northwest Tigray (79.3%) [26] and Northeast Ethiopia (98.0%) [14]. Whereas, it is higher than studies done in North Gondar (22.8%) [25] and Awi zone (39.8%) [13]. Since this study was conducted in remote and agrarian societies, which have no sufficient amount of vitamin k in the primary health care unit and also more than one-third of the women gave birth at home. Because of these, the magnitude of vitamin k injection at birth was extremely low.

### 4.2 Determinants of immediate newborn care practices

Though the level of optimal immediate newborn care practice is low, community and individual level factors block the utilization of the services. As result, this study found that the partner education level: mothers whose partners attended primary cycle school, high school and tertiary education were two times more likely to have optimal immediate newborn care practice. This finding is supported by evidence of systematic review and meta-analysis (SRMA) in Ethiopia [10], Gedeo Southern Ethiopia [33], Hossana town [28] and Enderta district [30]. This is because scientifically education increases the knowledge of households on health-seeking behaviors, empowering women to decide on the utilization of health services, particularly maternal, neonatal and child health services [9, 43]. Moreover, education is a tool that empowers the household to seek health services for newborns and pregnant women [9, 43]. Hence, mothers are more likely to receive optimal newborn care.

Antenatal care is an important entry point and creates a great opportunity for the utilization of maternal and neonatal health services [9]. In line with these, this study found that women who received the recommended ANC visit (≥4 visits) were three-fold times more likely to have optimal immediate newborn care practices. It is consistent with findings from Southwest Ethiopia [11], SRMA in Ethiopia [10], Southern Ethiopia [7], Nekemte city [15], Chancho district [27], Bahar Dar administrative city [29], Enderta district [30] and Gedeo Southern Ethiopia [33]. Moreover, this study found that pregnant women who initiate a 1st ANC visit between 4 and 6 months of gestational age and after 6 months of gestational age were less likely to have an optimal immediate newborn care practice by 53% and 89% respectively. This is because women delay the initiation of ANC visits according to World Health Organization (WHO) recommendation, they do not get adequate information and counseling services on the importance of maternal, neonatal and child health services and also the probability of practicing and utilizing essential newborn care are ignored or subside.

In line with WHO recommendations, all pregnant women should routinely receive iron and folic acid (IFA) supplementation during pregnancy together with appropriate dietary advice to prevent anemia [44]. This intervention is not only for the prevention of anemia but also increases the satisfaction level of the client on maternal and newborn health services and promotes health-seeking behaviors. Thus, this study found that mothers who received iron and folic acid (IFA) supplementation during pregnancy were three times more likely to have optimal immediate newborn care practices.

Women’s empowerment and husband decision-making in health care seeking enhance their decision-making authority regarding health-seeking behavior of mothers and newborns after delivery [9, 45]. Beside these, this finding reveals that women who decide on health services seeking and women whose husbands decide on health services seeking were two times more likely to have an optimal immediate newborn care practice. This is because women have a right to get adequate information on the benefit of maternal and newborn health services and also practice health-seeking behaviors when they have the autonomy to decide on health-seeking.

### 4.3 Impact of continuum of care on immediate newborn care practices

According to WHO directives, maternal health services are the main strategies for reducing neonatal mortality by advising pregnant women on how to access essential newborn care practices and educating them to care for their newborns when they visit health facilities and during community health worker visits to their homes [46]. Hence, the completion of a continuum of care in maternal health services is a big opportunity to improve the utilization of optimal immediate newborn care practice. Accordingly, this study found that completion of a continuum of care in maternal health services via both time and space dimensions are more likely to have effects on the utilization of optimal immediate newborn care practice. This finding is consistent with studies done in the Enderta district [30] and East Africa [32]. This is because, during ANC visits, health facility delivery and postnatal visit, mothers get adequate information and counseling on newborn care practice and the probability of getting optimal immediate newborn care practice increases [46]. Moreover, community health workers encourage mothers to practice optimal newborn care during home visiting. Hence, the utilization of maternal health services are increasing the key newborn care practices: early and exclusive breastfeeding, delayed bathing and cord care [47].

### 4.4 Limitation of study

The limitation of this study was some medical terms were difficult to translate exactly to local languages, which might have affected the respondents’ understanding. To reduce this limitation, local language experts translated the instruments and also data collectors, who were fluent in local languages and familiar with local terms, collected the data. Another limitation of this study was mothers who had a stillbirth were not comfortable to respond the questions properly. To overcome this inconvenience, they were interviewed a week after the event, which might have recall bias. Moreover, the health facility data were collected and recorded by health professionals, which might lead to social desirability bias.

## 5 Conclusion

This study found that magnitude of optimal immediate newborn care practices was low. Accordingly, women’s education status, receiving the recommended ANC visits, iron and folic supplementation (IFA) during pregnancy, women’s and husband’s decision-making power on health-seeking and early initiation of ANC visits within the recommended schedule were determinant factors for utilization of optimal immediate newborn care practices. As treatment effect, pregnant women who had received 1st ANC visit, completed recommended ANC visits, skilled delivery services, completed continuity of recommended ANC visits and skilled delivery service, completed a continuum of care in maternal health services via time dimension, completed key services of ANC package, completed key services of PNC services, completed whole key services of maternal health services package and completed a continuum of care in maternal health services via space dimension were significantly improved utilization of optimal immediate newborn care practice. Therefore, strengthening and increasing the completion rate of a continuum of care in maternal health services, improving iron and folic acid (IFA) supplementation program and household decision-making power on health-seeking behavior are strongly recommended to enhance the utilization of optimal immediate newborn care practices and also to reduce neonatal death and illness in the region.

## Declarations

### Author contribution statement

Muluwas Amentie Zelka: Conceived and designed the experiments; Performed the experiments; Analyzed and interpreted the data; Wrote the paper.

Alemayehu Work Yalew, Gurmesa Tura Debelew: Conceived and designed the experiments; Analyzed and interpreted the data; Wrote the paper.

### Funding statement

This work was supported by Addis Ababa University.

### Data availability statement

Data will be made available on request.

### Declaration of interest’s statement

The authors declare no conflict of interest.

### Additional information

No additional information is available for this paper.

## Appendix A Supplementary data

Questionnaires for Research Work Questionnaires for Research Work
