# Supplementary material for: Effects of continuity of maternal health services on immediate newborn care practices, Northwestern Ethiopia: multilevel and propensity score matching (PSM) modeling
Source: Heliyon. 2022 Dec 2;8(12):e12020. doi: 10.1016/j.heliyon.2022.e12020 (PMC9720007; doi:10.1016/j.heliyon.2022.e12020)
Supplement: Questionnaires for Research Work [file mmc1.docx]

## Annex – 1:- Research instrument

***Annex 1.1. Census format to enumerate pregnant women***

**Addis Ababa University**

**College of Health Science**

**School of Public Health**

**Research title:** Level and determinants of continuum of care in maternal health service and its effects on pregnancy and neonatal health outcome in Benishangul Gumuz Regional State, Northwestern Ethiopia.

**Investigators:** Mr. Muluwas Amentie, Prof. Alemayehu Worku and Dr Gurmesa Tura

**Part I – Participant Information sheet**

Informed consent for respondents providing information during census to identify pregnant women

**Introduction:**

Good morning/afternoon! My name is _________________. I am working with Mr. Muluwas Amentie who is currently conducting his PhD research and his research team presented in Addis Ababa University and Jimma University who are frequency following our work. We are visiting all households in the selected clusters/“*kebeles*” in the Benishangul Gumuz region to identify pregnant women for the study.

**Purpose of the census**

The purpose of this visit is simply to identify and register pregnant women who will be potentially the participants of the study.

**Procedures**

Among the region “*kebeles*”; 53 clusters/“*kebeles*” are scientifically selected in order to conduct a research on pregnant women. So, we are visiting all the households from the selected kebeles and communicate with respondents. It may take about 10 minutes of your time to speak to me and tell me the list of all female 15-49 years of age and some very few related questions.

**Risks and discomfort**

May I ask you about marital status and pregnancy status of the women, which may be personal information and may not be comfortable. But, they are very crucial for the study and important to improve maternal and child health services in the study area, you may fell free and tell me the information.

**Benefits**

Your participation will help us to find out more information on maternal health services and neonatal health condition which helps for further study. Now we will conduct study that intends to address maternal and neonatal health problems occurring during pregnancy, child birth and after birth. But we will not pay you any things and no direct benefit to you.

**Confidentiality**

The information you will give me will be kept confidential. The names you will provide me will be replaced with some codes and we will not communicate to someone else. No any names will be included during the interview and the data to be collected will also be used in aggregated form.

**Right to refuse or withdraw**

You have right to refuse or stop to answer any question to which you are not comfortable. However, your genuine information will have paramount importance in improving maternal health services in future.

Are you willing to provide me the information: 1. Yes, tick and proceed. 2. No, tick and Stop and thank them

Census conductor (enumerator):

Name ________________________, sig. _________, Date __________

Supervisor:

Name ________________________, sig. _________, Date __________

1. **General background information**

- Zone/Town Adm.: _______________.
- Woreda: _______________________.
- Kebele/Ketena: __________________.
- Got: ______________
- Name of head of the household: ________________________.
- Name of nearest health facility: _________________________.

1. **List of all 15 – 49 years old women and their pregnancy information**

| **S.No** | **Name of females in HH** (15 – 49years old) | **Age** | **Relation to head of HH** | **Marital Status** | **Pregnancy status** | **Approx. GA in wks** | **Set serial no for pregnant woman*** | **Remarks** |
| --- | --- | --- | --- | --- | --- | --- | --- | --- |
|  |  |  |  |  |  |  |  |  |
|  |  |  |  |  |  |  |  |  |
|  |  |  |  |  |  |  |  |  |
|  |  |  |  |  |  |  |  |  |
|  |  |  |  |  |  |  |  |  |
|  |  |  |  |  |  |  |  |  |
|  |  |  |  |  |  |  |  |  |
|  |  |  |  |  |  |  |  |  |
|  |  |  |  |  |  |  |  |  |
|  |  |  |  |  |  |  |  |  |

***** *Once a pregnant woman gets a serial number during the census, she is a potential candidate for the survey being study subject*

1. **Pregnancy screening checklist**

This checklist adapted from training and reference guide for a screening checklist to identify whether women are pregnant or not ([90](#_ENREF_90)). *Ask the following questions for all women whose age 15 – 49 years old and started a sexual experiences: In order to indicate eligibility women for the study.*

| **S. No** | **Probing question to indicating eligibility of pregnancy** | **Responses** | **Decision** |
| --- | --- | --- | --- |
|  | Are you known pregnant? (Confirmed at health facility or big abdomen with visible pregnancy) | 1. Yes 2. No | If “yes” **🡺 Eligible**  If “No” **🡺 Q_2_** |
|  | Did you have a baby less than 6 months ago? | 1. Yes 2. No | If “yes” **🡺 Non-eligible**  If “No” **🡺 Q_3_** |
|  | Are you fully or nearly-fully breastfeeding a baby < 6 months old and have you had no menstrual period since giving birth? | 1. Yes 2. No | If “yes” **🡺 Non-eligible**  If “No” **🡺 Q_4_** |
|  | Have you abstained from sexual intercourse since your LMP or delivery? | 1. Yes 2. No | If “yes” **🡺 Non-eligible**  If “No” **🡺 Q_5_** |
|  | Have you had a baby in the last 4 weeks? | 1. Yes 2. No | If “yes” **🡺 Non-eligible**  If “No” **🡺 Q_6_** |
|  | Did your last menstrual period start within the past 7 days | 1. Yes 2. No | If “yes” **🡺 Non-eligible**  If “No” **🡺 Q_7_** |
|  | Did your last menstrual period start within the past 12 wks | 1. Yes 2. No | If “yes” **🡺 Non-eligible**  If “No” **🡺 Q_8_** |
|  | Have you had a miscarriage or abortion in the past 7 days | 1. Yes 2. No | If “yes” **🡺 Non-eligible**  If “No” **🡺 Q_9_** |
|  | Have you had a miscarriage or abortion within the past 12wks | 1. Yes 2. No | If “yes” **🡺 Non-eligible**  If “No” **🡺 Q_10_** |
|  | Have you been using a reliable contraceptive method consistently and correctly? | 1. Yes 2. No | If “yes” **🡺 Non-eligible**  If “No” **🡺 Eligible** |

***Annex – 1.2: Baseline survey questionnaire***

**Addis Ababa University, College of Health Science, School of Public Health**

***Annex – 2.2.1: Informed Consent***

**Research title:** Level and determinants of continuum of care in maternal health service and its effects on pregnancy and neonatal health outcome in Benishangul Gumuz Regional State, Northwestern Ethiopia.

**Investigators:** Mr. Muluwas Amentie, Prof. Alemayehu Worku and Dr Gurmesa Tura

This informed consent consist of two parts: *information sheet* and *certificate of consent form*

***Part – I: Participant information Sheet***

**Introduction:**

Greeting, how are you my dear? My name is ___________ and I am working with Mr. Muluwas Amentie; who came from Addis Ababa University, College of Health Science which is currently carrying out his PhD research on maternal health services in Benishangul Gumuz Regional state, within the fairly scientifically sampled districts and clusters/“*kebeles”*.

**Purpose of the study**:

Maternal and neonatal mortality and morbidity are one of major public health issue in developing and low-income countries including Ethiopia. However, continuum of care in maternal health services is key strategies for reduction of maternal and neonatal mortality and morbidity. Thus, the aim of this study is measure level and determinants of continuum of care in maternal health services and its effects on pregnancy and neonatal health outcome.

**Procedure and participation:**

The method of this research is community and health facility linked prospective cohort study design. Pregnant women who are eligible for the study in some villages will be selected and follow up for 9 months in order to assess maternal health services and neonatal health issue. During the follow up period, we gather information during pregnancy time four times; during delivery time once and during postnatal period four time. So, your commitment in voluntary providing information is very vital for our achievement and future health program planner.

At the end of the follow up period, we will check how well the maternal health services up take will promote maternal and neonatal health in your community by comparing to other community that did not receive maternal health services. The information obtained in this survey will be useful to prepare and design strategies, programs and policy on maternal health issues.

As part of this study, we are collecting your information on socio-demographic, obstetric, pregnant related problems, pregnancy outcome, neonatal health status, maternal health care service utilization, neonatal mortality and morbidity. We are also collecting this information to measure a significant change on pregnancy outcome and neonatal health outcome contributed by being a mother completing continuum of care in maternal health services comparing with mother who were discontinuous care of maternal health services or never use maternal health service totally. This information enables the government and other stakeholders to develop programs to improve maternal health services. The interview will be conducted in phase by phase and it will take about 30 minutes per each phase. Be assured that your name will not be recorded and any other identifying information will be kept confidential and will not be shared with anyone else without your consent. Your participation is voluntary and you have the right not to participate fully or partially. Your decision about not to participate is respected and will not affect the health care you would normally receive.

**­­Risks and Discomfort**

There might be slight discomfort to share some personal information. However, we do not wish this to happen and you may refuse to answer any of the questions if you feel uncomfortable.

**­Benefits**

The research does not have any financially, health services and capacity building benefits. But your participation will help us to find out more about maternal and neonatal health issue in the community and to give maternal health services to mothers in your villages. If our study shows that the uptake of continuum of care in maternal health services had a significant improvement on pregnancy outcome and neonatal health status, we will be able to better improve maternal health in other villages in this area and other part of the country. And also gave an input for designing a strategies and formulation of maternal and child health programs based on the recommendation.

**Incentives:** We will not pay you for taking part in this study. However, we will thank you for your participation.

**Confidentiality**

The information that we collect in this study will be kept confidential. Any paper containing your name which will need to be kept for us to contact you will be kept under lock and will not be given to anyone except the investigators.

**Right to refuse or withdraw**

If you do not have to take part in this research, you have a full right to with draw from the study and refuse to participant in the study. Being refusing to participate and withdraw from the study will not affect your future treatment at the health facility or elsewhere in any way. You may stop participating in the interview at any time.

**Who to contact**

The study participant has the right to ask information or question on the unclear ideas about the research context or contents before or during the research work. If you have any questions you may ask now or later, you wish to ask questions later, you may contact: Mr. Muluwas Amentie, who was learning from Addis Ababa University and working at Assosa University, Telephone: *0913400353*. If you need further information beyond principal investigator, you can contact the following people or institute:

- Addis Ababa University College of Health Sciences IRB Secretary Office Tel: 0115512876
- Prof. Alemayehu Worku, School of Public Health, College of Health Sciences, Addis Ababa University; Mobile: 0911405652
- Dr. Gurmesa Tura, Department of Population and Family Health, College of Public Health and Medical Sciences, Jimma University; Mobile: 0912061646

Therefore, you may stop the interview at any time. Do you have any questions on what we talked so far? Now, do you agree to participate in the survey? Yes_____ No ______If, no respect the decision and thank her. If yes continue the interview.

**Name of the supervisor……………………. Signature………. Date……………..**

**Part – II: Certificate of Consent form**

**Title of research:** “Level and determinants of continuum of care in maternal health services and its effects on pregnancy and neonatal health outcome in Benishangul Gumuz Regional State, Northwestern Ethiopia”

I, the undersigned have been well aware of this research undertaking a post graduate degree partial fulfillment of research dissertation which supported and coordinated by AAU School of Public Health and designated by principal investigator Mr. Muluwas Amentie. I have fully informed in the language I understand about the research work.

I have been informed that all the information I shall provide to the interview will be kept confidential. I understood the research work has no any risk and no compensation. I have had the opportunity to ask questions on unclear about the research before and during the research work and to contact concerned bodies. I had a right to withhold information, skip questions to answer or have been answered to on the base of my satisfaction. I consent voluntarily to be a participant in this study and understand that I have the right to withdraw from the interview at any time without in anyway affecting my right. I also agreed about the confidentiality of the responses to be at a higher possible level.

I have read this form or it has been read to me in the language I understood the condition stated above, therefore, I am will and confirm my participation by signing the consent.

Agree to participate in the study: 1. Yes 2. No. (Mark one of them for verbal consent

**Signature/Finger print of Participant**: ___________________

***For illiterate person***; Name of witness signature: _____________________ (any 3^rd^ person)

Signature: _________________.

Date: ____________________

**Signature of the interviewer:**

Name ________________ Signature ________________ date _______________

**Signature of Supervisors:**

Name ________________ Signature ________________ date _______________

**Instruction:** *This part of questionnaire will be fulfill at the beginning of survey when the participants are candidate for the cohort study and please fill serial number of client which written in registration book unless this data is invalid.*

**Serial Number of Client: _________.**

**I. Socio-Demographic Characteristics of Respondents**

| **S/N** | **Questions** | **Responses** | **Code** | **Remark** |
| --- | --- | --- | --- | --- |
| Q_101_ | Household address | 1. Zone _________________________ 2. Woreda/sub city _________________ 3. Kebele/Ketena __________________. 4. HDA team _____________________. 5. Household number _______________. |  |  |
| Q_102_ | Place of residence | 1. Urban 2. Rural |  |  |
| Q_103_ | What is your age? | _________Years |  | In Completed year |
| Q_104_ | What is your religion? | 1. Orthodox 2. Muslim 3. Protestant 4. Catholic 5. Traditional belief (“*Musa*”) 6. Other, Specify _____ |  |  |
| Q_105_ | What is your ethnicity? | 1. Berta 2. Amhara 3. Oromo 4. Gumuz 5. Shinasha 6. Other, specify _________ |  |  |
| Q_106_ | What is your marital status? | 1. Married 2. Single 3. Divorced 4. Widowed |  |  |
| Q_107_ | What is your educational status? | 1. Illiterate 2. Read and write 3. Grade completed--------- |  |  |
| Q_108_ | What is your husband educational status? | 1. Illiterate 2. Read and write 3. Grade completed--------- |  |  |
| Q_109_ | What was your occupation during this pregnancy? | 1. House Wife 2. Gov’t employed 3. Merchant 4. Student 5. Other, specify______ |  |  |
| Q_110_ | What is your husband’s current occupation | 1. Government employee 2. Private employee 3. Self employee 4. Farmer 5. Other (specify)________ |  |  |
| Q_111_ | Do you have any information on maternal health services? | 1. Yes 2. No |  |  |
| Q_112_ | If “yes” for Q_111_, where is the source of information for ANC service? | 1. Radio 2. TV 3. Other (specify)­­­­­­______________ |  |  |
| Q_113_ | If “yes” for Q_111_, where is the source of information for delivery service? | 1. Radio 2. TV 3. Other (specify)______________ |  |  |
| Q_114_ | If “yes” for Q_111_, where is the source of information for postnatal service? | 1. Radio 2. TV 3. Other (specify)______________ |  |  |
| Q_115_ | Accessibility of health facility (distance between their kebele to the nearest health facility) | 1. Health post ______km (____ minutes by foot) 2. Health center ______km (____ minutes by foot) 3. Hospital ______km (____ minutes by foot) |  |  |
| Q_116_ | Is there maternal health services (ANC, skilled delivery and PNC services) in the nearest health facility | 1. Yes 2. No |  |  |
| Q_117_ | If “yes” for Q_117_; did the health facility provide maternal health services for the community? | 1. Yes 2. No |  |  |
| Q_118_ | What was your household monthly income in this year? | 1. _______Birr(Ethio. birr) 2. No any income 3. I don’t know |  |  |
| Q_119_ | What is the main source of drinking water for members of your household | 1. Pipe water  2. Public hand pump  3. Public tap/standpipe (‗Bono‘)  4. Protected dug well  5. Unprotected dug well  6. Protected spring  7. Unprotected spring  8. Rain water  9. Other (specify) __________________ |  |  |
| Q_120_ | What kind of toilet facility do you have that your household members use? | 1. Flush toilet  2. Traditional Pit toilet/latrine  3. Ventilated Improved Pit (VIP) latrine  4. No facility-use open field  5. Other (specify) _________ |  |  |
| Q_121_ | How is your residential home ownership? | 1. Own home  2. Rented home  3. Other (specify) _______________ |  |  |
| Q_122_ | How many rooms/classes does your household have? | _________ rooms |  |  |
| Q_123_ | How many rooms in this household are used for sleeping | _________ rooms |  |  |
| Q_124_ | Do you have a separate room which is used as a kitchen? | 1. Yes 2. No |  |  |
| Q_125_ | What is the main material of the floor of the home?  **(Record by observing)** | 1. Natural floor- earth 2. Natural floor-dung 3. Rudimentary floor with wood/bamboo 4. Finished floor with Cement 5. Other (specify) ______ |  |  |
| Q_126_ | What is the main material of the roof of the home?  **(Record by observing)** | 1. Thatched 2. Corrugated iron sheet 3. Other (specify) ______ |  |  |
| Q_127_ | What is the main material of the wall of the home?  **(Record by observing)** | 1. No wall 2. Wood without mud 3. Wood with mud 4. Wood with cement covered 5. Cement blocks or Bricks 6. Other (specify) ____________ |  |  |
| Q_128_ | What type of fuel do you mainly use for cooking in your household?  **(Multiple response is possible)** | 1. Electricity 2. Natural gas 3. Biogas 4. Kerosene 5. Charcoal 6. Firewood 7. Dung 8. Other (specify) ___________ |  |  |
| Q_129_ | Does your household own the following?  **(Multiple response is possible)** | 1. Electricity 2. Radio 3. Television? 4. A landline telephone functioning 5. Refrigerator |  |  |
| Q_130_ | Does any member of your household own the following?  **(Multiple response is possible)** | 1. Watch 2. Mobile phone 3. Bicycle 4. Motor cycle 5. A car or truck 6. Animal drawn cart |  |  |
| Q_131_ | Does any member of this household own any agricultural land? | 1. Yes 2. No | If none enter 0 (zero) | |
| Q_132_ | If “yes” for Q130, how many hectares? | _____________hectares |  |  |
| Q_133_ | Does this household own any livestock, herds, other farm animals, or poultry? | 1. Yes 2. No |  |  |
| Q_134_ | If “yes” for Q132, how many  **(Multiple response is possible)** | 1. Cattle: ­­­­­­________________ 2. Milk cows or bulls: ____________ 3. Horses: ______________ 4. Donkeys or mules: _________ 5. Goats : __________ 6. Sheep: __________ 7. Chickens: __________ | If none enter 0 (zero) | |

**II. Obstetric characteristics and maternal health care service practice**

| **S/N** | **Questions** | **Responses** | **Code** | **Remark** |
| --- | --- | --- | --- | --- |
|  | **Past obstetric history and maternal health services for current pregnancy** | | | |
| Q_201_ | What was your age at first marriage? | ________years (write in completed years) |  |  |
| Q_202_ | What was your age at your first pregnancy? | ________years (write in completed years) |  |  |
| Q_203_ | Including this pregnancy; how many pregnancies have you ever had? | 1. _______ pregnancies 2. No. of live births_______ 3. No. of stillbirths _______ 4. No. of abortions_______ 5. ***If she is primigravida*** | ***Go to _Q215_*** | |
| Q_204_ | Have you ever had abortion? | 1. Yes 2. No |  |  |
| Q_205_ | If “yes” to Q_204_, how many abortions? | ___________ |  |  |
| Q_206_ | Place of delivery for previous pregnancy? | 1. Home 2. Health post 3. Health center 4. Hospital 5. Other specify _____ |  |  |
| Q_207_ | Who attended/assisted your previous delivery? | 1. Family/Friend/relative 2. TBA 3. HEW 4. Midwife/nurse/Ho 5. Medical doctor/Physician 6. Other (specify) __________ |  |  |
| Q_208_ | If she has delivered, what was the mode of delivery for previous pregnancy? | 1. Spontaneous vaginal delivery 2. operative vaginal delivery (Vacuum/forceps) 3. Destructive vaginal delivery for dead fetal 4. Operative abdominal delivery (C/S) |  |  |
| Q_209_ | Have you ever had any pregnant related problems before this pregnancy? | 1. No 2. Yes 3. I didn’t known |  |  |
| Q_210_ | If “yes” for Q_209,_ which of the following pregnant related problems did you suffered?  **(Multiple response is possible)** | 1. Vaginal bleeding 2. Severe headache 3. Severe abdominal pain 4. Drowsiness 5. Facial swelling 6. Hand swelling 7. Persistent vomiting 8. Others (specify)__________ |  |  |
| Q_211_ | Have you faced any pregnancy related health problems during labour or childbirth before this pregnancy? | 1. No 2. Yes 3. I didn’t known |  |  |
| Q_212_ | If “yes” for Q_211_, which of the following problems did you suffered?  **(Multiple response is possible)** | 1. Premature rupture of membranes 2. Preterm labour 3. Obstructed labour 4. Elevated blood pressure 5. Excessive bleeding during labour 6. Intrauterine fetal death 7. Others (specify) ______________ |  |  |
| Q_213_ | Have ever use any maternal health services for previous pregnancy? | 1. Yes 2. No |  |  |
| Q_214_ | If “yes” for Q_213_; which component of maternal health services did you received?  **(Multiple response is possible)** | 1. 1^st^ ANC visit services 2. 4^th^ or ANC visit services 3. Institutional delivery 4. 1^st^ PNC visit services 5. 4^th^ PNC visit services |  |  |
|  | **Present obstetric history and maternal health services for current pregnancy** | | | |
| Q_215_ | Date of LMP of this pregnancy | _­_____days / _______month/______year |  |  |
| Q_216_ | How long was this pregnancy in month? | _________Months |  |  |
| Q_217_ | Have you ever had any pregnant related problems during this pregnancy? | 1. No 2. Yes 3. I didn’t had any pregnancy before 4. I didn’t known |  |  |
| Q_218_ | If “yes” for Q_217,_ which of the following pregnant related problems did you suffered?  **(Multiple response is possible)** | 1. Vaginal bleeding 2. Severe headache 3. Severe abdominal pain 4. Drowsiness 5. Facial swelling 6. Hand swelling 7. Persistent vomiting 8. Others (specify)__________ |  |  |
| Q_219_ | Have you ever use any ANC services during pregnancy? | 1. Yes 2. No |  |  |
| Q_220_ | If “yes” for Q_219_; which component of ANC services did you received?  **(Multiple response is possible)** | 1. 1^st^ ANC visit services 2. 2^nd^ ANC visit services 3. 3^rd^ ANC visit services 4. 4^th^ or ANC visit services |  |  |
| Q_221_ | Do you plan to use any maternal health services from health facility? | 1. Yes 2. No |  |  |
| Q_222_ | If “No” for Q_221_; what are the reason for not to plan to use maternal health services at health facility? | 1. I don’t know about services 2. No problems encountered 3. Health institution was too far 4. No money to pay for service 5. No transportation 6. Can’t pay for transportation 7. Influence of other peoples 8. Fear of lack of privacy 9. Male health professionals 10. Other (specify)_____________ |  |  |

***Annex – 1.2: Registration format: Continuity of Maternal Health Services, Maternal and Neonatal Health Condition***

**Addis Ababa University, College of Health Science, School of Public Health**

- - Woreda (District) ________________ Kebele (Name) _____________
  - Kebele distance from the nearest health post ______km (____ minutes walking distance)
  - Kebele distance from the nearest health center ______km (____ minutes walking distance)
  - Kebele distance from the nearest hospital ______km (____ minutes walking distance)

| Serial number | Data of Last Menstrual Period (**LMP**) | BMI of mother | Services received during 1^st^ ANC visit (**A**) | Services received during 2^nd^ ANC visit (**B**) | Services received during 3^rd^ ANC visit (**C**) | Services received during 4^th^ or more ANC visit (**D**) | Any illness during this pregnancy (**1 = yes 2 = No**) | If “yes”, what are those illness/problems (**E**) | Pregnancy outcome: ***1= abortion, 2=Stillbirth or 3=Alive birth*** | Mode of delivery (**F**) | Place of birth for the current birth (**G**) | Who helped the current birth (**H**) | Have you been referred (**1 = yes; 2 = No**) | Is there any problems/illness in this birth (**1 = yes; 2 = No**) | If “yes”, what are those illness/problems (**I**) | How many newborn born in this birth ( **1 = Single 2 = Twin**) | APGAR score of newborn | Birth weight of the newborn | Head circumference of the newborn | Length of the newborn | Any services/care offered for the newborn immediately(**J)** | What was the fetal condition at birth (**K**) | Services received during 1^st^ PNC visit (**L**) | Services received during 2^nd^ PNC visit (**M**) | Services received during 3^rd^ PNC visit (**N**) | Services received during 4^th^ PNC visit (**O**) | Is there continuum of care (CoC) in MHS (**1 = yes 2 = No**) | Mother condition up to 42 days after birth (**P**) | If mother “died/illness”, what are the main cause or S/S (**Q**) | If mother “died/illness”, what was the time of death (**R**) | Condition of alive birth after birth up to 28days (**S**) | If newborn “died”, what are the main cause or S/S (**T**) | If newborn “died”, at what days does it occur |
| --- | --- | --- | --- | --- | --- | --- | --- | --- | --- | --- | --- | --- | --- | --- | --- | --- | --- | --- | --- | --- | --- | --- | --- | --- | --- | --- | --- | --- | --- | --- | --- | --- | --- |
|  |  |  |  |  |  |  |  |  |  |  |  |  |  |  |  |  |  |  |  |  |  |  |  |  |  |  |  |  |  |  |  |  |  |
|  |  |  |  |  |  |  |  |  |  |  |  |  |  |  |  |  |  |  |  |  |  |  |  |  |  |  |  |  |  |  |  |  |  |
|  |  |  |  |  |  |  |  |  |  |  |  |  |  |  |  |  |  |  |  |  |  |  |  |  |  |  |  |  |  |  |  |  |  |
|  |  |  |  |  |  |  |  |  |  |  |  |  |  |  |  |  |  |  |  |  |  |  |  |  |  |  |  |  |  |  |  |  |  |

**Description and coded choices**

1. **Services received: 1^st^ ANC**

1 = informed sign of pregn complication

2 = Blood pressure measured

3 = Urine sample taken

4 = Blood sample taken

5 = Received Nutrition Counseling

6 = Iron folate supplementation

7 = Protection of birth from tetanus

1. **Services received: 2^nd^ ANC**

1 = informed sign of pregn complication

2 = Blood pressure measured

3 = Urine sample taken

4 = Blood sample taken

5 = Received Nutrition Counseling

6 = Iron folate supplementation

7 = Protection of birth from tetanus

1. **Services received: 3^rd^ ANC**

1 = informed sign of pregn complication

2 = Blood pressure measured

3 = Urine sample taken

4 = Blood sample taken

5 = Received Nutrition Counseling

6 = Iron folate supplementation

7 = Protection of birth from tetanus

1. **Services received: 4^th^ ANC**

1 = informed sign of pregn complication

2 = Blood pressure measured

3 = Urine sample taken

4 = Blood sample taken

5 = Received Nutrition Counseling

6 = Iron folate supplementation

7 = Protection of birth from tetanus

1. **Illness during pregnancy**

1 = excessive bleeding/APH

2 = Anemia

3 = Fever

4 = excessive vomiting

5 = Weight loss

6 = Preeclampsia/eclampsia

7 = others (specify)

1. **Mode of delivery**

1 = Normal labor (vaginally)

2 = Vaginal instrument

3= Caesarean

1. **Place of delivery**

1 = Home

2 = Health post

3 = Health center

4 = Hospital

5 = Private clinic

1. **Who helped the birth?**

1 = Traditional birth attendant

2 = Family (friend)

3 = HEW

4 = HW (midwife, nurse)

1. **Illness during delivery**

1 = excessive bleeding

2 = Prolonged labour

3 = Obstructed labour

4 = Trauma/tear of birth canal

5 = others (specify)

1. **Any care offered for newborn**

1= Dry and stimulate the baby

2 = Keep the baby warm by skin to skin

3 = Appropriate cord care

4 = initiate breast feeding within 1hr birth

5 = Vitamin K injection

1. **Fetal condition at birth**

1 = Alive and normal

2 = Alive but malformed

3 = Stillbirth

4 = Abortion

1. **Services received: 1st PNC**

1= Physical examination

2= Counseling on breastfeeding

3 =Contraceptives

4= Blood test for anemia

5= Nutritional supplements

6= Information on warning signs problems

1. **Services received: 2^nd^ PNC**

1= Physical examination

2= Counseling on breastfeeding

3 =Contraceptives

4= Blood test for anemia

5= Nutritional supplements

6= Information on warning signs problems

1. **Services received: 3^rd^ PNC**

1= Physical examination

2= Counseling on breastfeeding

3 =Contraceptives

4= Blood test for anemia

5= Nutritional supplements

6= Information on warning signs problems

1. **Services received: 4^th^ PNC**

1= Physical examination

2= Counseling on breastfeeding

3 =Contraceptives

4= Blood test for anemia

5= Nutritional supplements

6= Information on warning signs problems

1. **Mother condition up to 42**

1 = Died (mortality)

2 = Ill/sick (morbidity)

3 = Wellbeing

1. **Main cause or S/S**

1 = Bleeding

2 = Fever (infection)

3 = Convulsion

4 = others (specified)

1. **Time of death**

1 = during pregnancy

2 = during labor/delivery

3 = during postnatal period

1. **Condition of alive birth (28days)**

1 = Died (mortality)

2 = Ill/sick (morbidity)

3 = Wellbeing

1. **The main cause or S/S death**

1 = Birth Aspexia

2 = Preterm

3 = Low birth weigh

4 = Neonatal tetanus

5 = Hypothermia

6 = Diaharrial diseases

7 = Fever/malaria

7 = Pneumonia

8 = Measles

9 = Others (specify)

***Annex – 1.3: Continuum of care in maternal health care services***

**Instruction:** *This questionnaire will be fulfilling at the end of postnatal period and please fill serial number of client which written in registration book unless this data is invalid*

**Serial Number of Client: _________.**

| **S. No** | | **Questions and filters** | | **Coding categories** | | | **Skip to** | | | **Remark** | |
| --- | --- | --- | --- | --- | --- | --- | --- | --- | --- | --- | --- |
| Filter | | *Only ask the questions in this section when the woman came to the health facility or at home level start receiving maternal health services during pregnant, labour or after delivery within 42 days.* | | | | | | | | | |
|  |  | ***I’d like to ask you some questions about the woman’s health during her pregnancy and ANC uptake*** | | | | | | | | | |
| Q_501_ | | Have you attended ANC for your last pregnancy? | | | 1. Yes______ 2. No_______ | | |  | |  | |
| Q_502_ | | If “*yes*” for Q_501;_ Why did she first seek ANC service | | | 1. Only for check up 2. Because of the presence of problems 3. I didn’t know | | |  | |  | |
| Q_503_ | | If “*due to presence of problems*” for Q_502_; which problem did she first seek antenatal care? (specified the problems by verbatim) | | | _______________________________________________________________. | | |  | |  | |
| Q_504_ | | If “*No*” for Q_501_, Why didn’t you attend ANC visit?  (**Multiple answers are possible)** | | | 1. I don’t know about ANC 2. No problems encountered 3. Health institution was too far 4. No money to pay for service 5. No transportation 6. Can’t pay for transportation 7. Influence of other peoples 8. Fear of lack of privacy 9. Male health professionals 10. Other (specify)_____________ | | |  | |  | |
| **Ask questions 505-516 only for women who attend ANC for their current pregnancy**. | | | | | | | | | | | |
| Q_505_ | Did she receive recommended all ANC visit during this pregnancy period? (*starting from 1^st^ visit to 4^th^ visit*) | | | | 1. No 2. Yes | | |  | |  | |
| Q_506_ | If “*yes”* for Q_505_; which visit of ANC did you received?  (**Multiple answers are possible)** | | | | 1. 1^st^ visit of ANC services 2. 2^nd^ visit of ANC services 3. 3^rd^ visit of ANC services 4. 4^th^ visit of ANC services | | |  | |  | |
| Q_507_ | If “*yes”* for Q_505_; which key services or interventions were you received during ANC visit? | | | | 1. Informed sign of pregnant complications 2. Blood pressure measured 3. Urine sample taken 4. Blood sample taken 5. Nutritional counseling 6. Iron folate supplementation 7. Protection of birth from tetanus 8. Other (specify) ___________________ | | |  | |  | |
| Q_508_ | If “*no”* for Q_505_; Why did you lost to follow up attending ANC visit? (*starting from 1^st^ visit to 4^th^ visit*)  (**Multiple answers are possible)** | | | | 1. I don’t know about ANC 2. No problems encountered 3. Health institution was too far 4. No money to pay for service 5. No transportation 6. Can’t pay for transportation 7. Influence of other peoples 8. Fear of lack of privacy 9. Male health professionals 10. Other (specify)_____________ | | |  | |  | |
| Q_509_ | At what gestation age was her first ANC visit attended? | | | | 1. 1-3 months of pregnancy 2. 4-6 months of pregnancy 3. After 6 months of pregnancy | | |  | |  | |
| Q_510_ | Where were you attended? | | | | 1. At home 2. At clinic/health post 3. At health center 4. Hospital | | |  | |  | |
| Q_511_ | Who attended your ANC visits? | | | | 1. Doctor 2. Nurse/HO 3. H.E.W 4. TTBA 5. TBA 6. Other (specify)_____________ | | |  | |  | |
| Q_512_ | Would you paid for ANC service | | | | 1. Yes 2. No | | |  | |  | |
| Q_513_ | If “ *yes*” for Q_512_; how do you rate the cost of service | | | | 1. Very expensive 2. Expensive 3. Fair 4. Cheap 5. Very cheap | | |  | |  | |
| Q_514_ | Have you received information to deliver in health facilities? | | | | 1. Yes 2. No | | |  | |  | |
| Q_515_ | Have you had any health related problems during this pregnancy? | | | | 1. Yes 2. No | | |  | |  | |
| Q_516_ | If “*yes*” to Q_515_, Which of the following problems?  ***(Multiple answers are possible)*** | | | | 1. Vaginal bleeding 2. Severe Headache 3. Severe abdominal pain 4. Drowsiness 5. Facial swelling 6. Hand swelling 7. Persistent vomiting 8. No fetal movement after 24 weeks 9. Others (specify)__________ | | |  | |  | |
| ***I’d like to ask you some questions about the woman’s health during her pregnancy and uptake of delivery service*** | | | | | | | | | | | |
| Q_517_ | Where did you deliver your current childbirth? | | 1. In health facilities 2. At home 3. In forest | | |  | | | |  | |
| Q_518_ | If your response to Q_517_ is “at health facility”, What was the mode of delivery? | | 1. Spontaneous vaginal (SVD) 2. Assisted vaginal delivery (AVD) 3. Caesarean section (C/S) 4. Others (specify)___________ | | | *If home and forest delivery jump* ***Q_518_*** *and* ***Q_519_*** | | | | | |
| Q_519_ | If your response to Q_517_ is “at health facility”, Why you preferred to deliver in health facility? | | 1. Because of my previous bad experience from home delivery 2. I was informed to deliver in HFs 3. I have faced obstetric problems which forced me to deliver in health facility 4. Others (Specify)___________ | | |  | | | |  | |
| Q_520_ | Was the placenta delivered after child birth? | | 1. No 2. Yes | | |  | | | |  | |
| Q_521_ | If “yes” for Q_523_; at what time does it delivered? *Mention time in minute* | | ____________ minutes | | |  | | | |  | |
| Q_522_ | Is there retained placenta? | | 1. No 2. Yes | | |  | | | |  | |
| Q_523_ | Did you face any childbirth related health problems during labour and delivery? | | 1. Yes 2. No | | |  | | | |  | |
| Q_524_ | If “yes” to Q_523_, which of the following problems?  (**Multiple answers are possible)** | | 1. Premature rupture of membranes 2. Preterm labour 3. Obstructed labour 4. Elevated blood pressure 5. Excessive bleeding during labour 6. Intrauterine fetal death 7. Others (specify) ______________ | | |  | | | |  | |
| Q_525_ | If “yes” to Q_524_, did you receive any treatment or care for the problems encountered during child birth? | | 1. Yes 2. No | | |  | | | |  | |
| Q_526_ | If “yes” to Q_525_, where care or treatment was sought and obtained | | 1. Traditional healers 2. Health post/clinic 3. Health center 4. Hospital 5. Other (specify) ____________________ | | |  | | | |  | |
| Q_527_ | If “no” to Q_525_, what was the main reason why care was not sought? | | 1. Not knowing the impact of illness 2. Past good obstetric outcome at home 3. Not nearby health facility 4. Lack of transport 5. Lack of money 6. Other (specify) _____________________ | | |  | | | |  | |
| Q_528_ | How long was the duration of labour? | | 1. Less than half a day(<12 hours) 2. Between 12 – 24 hours 3. Greater than 24hours | | |  | | | |  | |
| Q_529_ | Have you faced any health problems immediately after delivery? | | - 1. No   2. Yes, specify them ___________________ | | |  | | | |  | |
| Q_530_ | Who made the final decision about your place of last delivery? | | Just me  My husband  My relatives  Other people, specify__________ | | |  | | | |  | |
| Q_531_ | Do you attend the whole service of ANC visit and institutional delivery? | | 1. Yes 2. No | | |  | | | |  | |
| Q_532_ | If “No” for Q_531_; what are the reason for not continuity of care from ANC service to institutional delivery? | | ____________________________________________________________________________________________________________. | | |  | | | |  | |
| **Ask questions 533 – 535 for women who gave their last birth at home only** | | | | | | | | | | | |
| Q_533_ | Why you preferred home delivery? | | 1. The labour was going well 2. I feel more comfortable at home 3. Close attention from relatives and family 4. It is my usual practice 5. Previous bad experience from ID 6. Cannot afford to pay for health services 7. No transportation services 8. Cannot pay for transportation services 9. Our culture doesn’t allow me ID 10. Other reasons, specify______________ | | |  | | | |  | |
| Q_534_ | During your home delivery; who attends your delivery? | | 1. Health Workers 2. TTBA 3. TBA 4. Relatives or family members 5. Other people, specify ________ | | |  | | | |  | |
| Q_535_ | Why did you prefer the above people to attend your delivery? | | Write the reasons _______________________. ______________________________________. | | |  | | | |  | |
|  | *I’d like to ask you some questions about the woman’s health after delivery up to 42 days (postnatal period) and uptake of postnatal care service* | | | | | | | | | | |
| Q_536_ | Does the nearest health facility give postnatal care services? | | 1. No 2. Yes | | | | | |  | |  |
| Q_537_ | Did you attend the recommended all visit of postnatal services within the six weeks after delivery? | | 1. No 2. Yes | | | | | |  | |  |
| Q_538_ | If “yes” for Q_537_; which visit of postnatal health services received?  (**Multiple answers are possible**) | | 1. 1^st^ visit of PNC services 2. 2^nd^ visit of PNC services 3. 3^rd^ visit of PNC services 4. 4^th^ visit of PNC services | | | | | |  | |  |
| Q_539_ | If “yes” for Q_537_; which postnatal services/key interventions did you receive when you went back to health facility after delivery?  (**Multiple answers are possible**) | | 1. Physical examination 2. Immunization of baby 3. Counseling on proper nutrition 4. Family planning services 5. Breast feeding education 6. Other (specify)________________ | | | | | |  | |  |
| Q_540_ | If “yes” for Q_537_; how many days after discharge from maternity or home delivery did you first see the health care provider for postpartum check-up? | | 1. __________days 2. I don’t know 3. I don’t remember | | | | | |  | |  |
| Q_541_ | If “yes” for Q_537_; why did you go for postnatal services?  (**Multiple answers are possible**) | | 1. Because of illness 2. Because the baby needed it’s immunization 3. Because the midwife had told me I should 4. Because I wanted to start family planning 5. Because I wanted to make sure I am back to normal 6. Other (specify) ____________________________ | | | | | |  | |  |
| Q_542_ | If “no” for Q_537_, What are the factors that prevented you from attending postnatal services?  (**Multiple answers are possible)** | | 1. Health professional shouted at me 2. They did not teach me well 3. Examined me roughly 4. Waiting more time at the facility 5. Religious forbidden 6. Ignorance of my privacy 7. Other (specify)_______________ | | | | | |  | |  |
| Q_543_ | Are there any cultural factors that prevent you from attending postnatal services? | | 1. No 2. Yes | | | | | |  | |  |
| Q_544_ | If “yes” for Q_543_, specified those cultural factors. | | _______________________________________________________________________ | | | | | |  | |  |
| Q_545_ | Did you experience any complications during postpartum period? | | 1. No 2. Yes 3. I don’t know | | | | | |  | |  |
| Q_546_ | If “yes” for Q_545_; which complications did she experienced during postpartum period?  (**Multiple answers are possible)** | | 1. Heavy bleeding 2. Loss of consciousness 3. Fever with or without chills 4. Foul smelling discharge 5. Convulsions/rigidity 6. Headache, visual disturbances 7. Severe abdominal pain 8. Other (specify)______________ | | | | | |  | |  |
| Q_547_ | Did the women who attend the whole service of ANC visit and institutional delivery service for the PNC service? | | 1. Yes 2. No | | | | | |  | |  |
| Q_548_ | If “no” for Q_547_; what are the reason for not continuum of care from ANC service and institutional delivery to PNC services? | | _______________________________________________________________________________________________________________________________. | | | | | |  | |  |
| ***Space dimension of continuum of care in maternal health services*** | | | | | | | | | | | |
| Q_549_ | Did you obtain any maternal health services at the home level by health professional? | | 1. Yes 2. No | | | | | |  | |  |
| Q_550_ | If “yes” for Q_549;_ which services did you receive  **(Multiple responses are possible)** | | 1. ANC services 2. Skilled delivery services 3. PNC services | | | | | |  | |  |
| Q_551_ | If “no” for Q_550;_  what are the main reasons | | ___________________________________________ | | | | | |  | |  |
| Q_552_ | Did the health worker link to the health facility during home visiting? | | 1. Yes 2. No | | | | | |  | |  |
| Q_553_ | If “yes” for Q_552;_ to which health facility did the linkage made? | | 1. Health post/clinic 2. Health center 3. Hospital | | | | | |  | |  |
| Q_554_ | If “no” for Q_552_; what are the main reasons for health workers did not link to the health facility | | 1. I am not volunteer to go to the health facility 2. My culture forbidden to go to the health facility 3. The health worker did not tell me any things 4. I belief that I am at normal condition 5. other (specify) ______________________________ | | | | | |  | |  |
| Q_555_ | Did maternal health services(ANC, Skilled delivery and PNC) offered at the community level by health workers, TBA, TTBA etc | | 1. Yes 2. No | | | | | |  | |  |
| Q_556_ | Did maternal health service integrated with other community services: malaria prevention, TT injection, Immunization at the community level | | 1. Yes 2. No | | | | | |  | |  |
| Q_557_ | Did the health work linked with community health support for maternal health services? | | 1. Yes 2. No | | | | | |  | |  |
| Q_558_ | Is there any community advocacy and sensitization on maternal health services? | | 1. Yes 2. No | | | | | |  | |  |
| Q_559_ | Is there any community support to improve maternal health services (ANC, Skilled delivery and PNC) within the community? | | 1. Yes 2. No | | | | | |  | |  |
| Q_560_ | If “yes” for Q_559_; mention support that offered for women | | ___________________________________________________________________________________________. | | | | | |  | |  |
| Q_561_ | If “no” for Q_560_; what are reasons why the community did not support? | | ____________________________________________________________________________________________. | | | | | |  | |  |

***Annex – 1.4: Neonatal health condition and their outcome***

**Instruction:** *This questionnaire will be fulfilling at the end of neonatal period (at one month after delivery) or event of death or morbidity occurred on the newborn child and please fill serial number of women which written in registration book unless this data is invalid*

**Serial Number of Client: _________.**

| **S. No** | **Questions and filters** | **Coding categories** | **Skip to** |
| --- | --- | --- | --- |
| Filter | *Only ask the questions in this section when newborn survive for one month after delivery) or at any time after delivery newborn child suffered with death or illness.*  ***I’d like to ask you some questions about the neonatal health condition*** | | |
| Q_601_ | What was the sex of your newborn? | 1. Male 2. Female |  |
| Q_602_ | What is the weight of newborn at birth? (***state in kilogram***) | _________ kilogram/s |  |
| Q_603_ | What is head circumference of the baby (cm) | __________cm |  |
| Q_604_ | What is the length of the baby (cm) | __________cm |  |
| Q_605_ | What is the APGAR score of newborn at birth | At first minute _________  At fifth minutes ________ |  |
| Q_606_ | At what gestational age (GA), did the baby born? | __________ weeks |  |
| Q_607_ | When did the amniotic member break? | 1. Before labour started 2. During labour 3. Unknown |  |
| Q_608_ | How many hours passed between her water breaking and birth? | ________________ hours |  |
| Q_609_ | What was the colour of water | 1. Clear 2. Yellow 3. Green 4. Brown 5. Dark red bright red 6. Unknown |  |
| Q_610_ | Did the water smell bad | 1. Yes 2. No |  |
| Q_611_ | Was the baby ever breast feed? | 1. Yes 2. No 3. I don’t know |  |
| Q_612_ | Did the baby cry immediately after birth | 1. Yes 2. No |  |
|  | ***Immediate new-born care practice*** | |  |
| Q_613_ | Did the baby receive any of the following care/service? | 1. Dry and stimulate the baby 2. Keep the baby warm by skin to skin 3. Appropriate cord care 4. initiate breast feeding within 1hr of birth 5. Vitamin K injection |  |
| Q_614_ | What was the health condition of the neonatal for this current birth? | 1. Alive and normal 2. Alive but having illness 3. Died/mortality |  |
| Q_615_ | Was the newborn able to breaths immediately after birth? | 1. Yes 2. No |  |
| Q_616_ | Was the newborn able to cry immediately after birth? | 1. Yes 2. No |  |
| Q_617_ | Did the newborn have any pulsation immediately after birth? | 1. Yes 2. No |  |
| Q_618_ | Did the newborn color is blue or pale immediately after birth? | 1. Yes 2. No |  |
| Q_619_ | Did the newborn muscle tone is limb (absence of movement at all) immediately after birth? | 1. Yes 2. No |  |
